# Unlocking Nature’s Secrets: Molecular Insights into Postharvest Pathogens Impacting Moroccan Apples and Innovations in the Assessment of Storage Conditions

**DOI:** 10.3390/plants13040553

**Published:** 2024-02-18

**Authors:** Mohammed Khadiri, Hassan Boubaker, Salah-Eddine Laasli, Abdelaaziz Farhaoui, Said Ezrari, Nabil Radouane, Mohammed Radi, Latifa Askarne, Essaid Ait Barka, Rachid Lahlali

**Affiliations:** 1 Phytopathology Unit, Department of Plant Protection, Ecole Nationale d’Agriculture de Meknès, Km10, Rte Haj Kaddour, BP S/40, Meknès 50001, Morocco; khadiri.eps@gmail.com (M.K.); laaslisalaheddine@gmail.com (S.-E.L.); farhaoui.aziz22@gmail.com (A.F.);; 2 Laboratoire de Biotechnologies Microbiennes et Protection des Végétaux, Faculté des Sciences, Université Ibn Zhor, BP 8106, Agadir 80000, Morocco; h.boubaker@uiz.ac.ma (H.B.); l.askarne@uiz.ac.ma (L.A.); 3 Department of Biology, Laboratory of Biotechnology and Valorization of Bio-Resources (BioVaR), Faculty of Sciences, Moulay Ismail University, BP 11201, Zitoune, Meknes 50000, Morocco; 4 Microbiology Unit, Laboratory of Bioresources, Biotechnology, Ethnopharmacology and Health, Faculty of Medicine and Pharmacy Oujda, University Mohammed Premier, Oujda 60000, Morocco; 5 African Genome Center, Mohammed VI Polytechnic University (UM6P), Ben Guerir 43150, Morocco; nabil.radouane@usmba.ac.ma; 6 Laboratory of Environment and Valorization of Microbial and Plant Resources, Faculty of Sciences, Moulay Ismail University, BP 11201, Zitoune, Meknes 50000, Morocco; 7 Induced Resistance and Plant Biosection Research Unit-EA 4707-USC INRAE1488, Reims Cham-pagne-Ardenne University, 51687 Reims, France

**Keywords:** apple, postharvest, storage, fungal pathogens, losses, Morocco

## Abstract

Apple production holds a prominent position in Morocco’s Rosaceae family. However, annual production can fluctuate due to substantial losses caused by fungal diseases affecting stored apples. Our findings emphasize that the pre-storage treatment of apples, disinfection of storage facilities, box type, and fruit sorting are pivotal factors affecting apple losses during storage. Additionally, the adopted preservation technique was significantly correlated with the percentage of damage caused by fungal infections. Blue mold accounts for nearly three-quarters of the diseases detected, followed by gray rot with a relatively significant incidence. This study has revealed several fungal diseases affecting stored apples caused by pathogens such as *Penicillium expansum*, *Botrytis cinerea*, *Alternaria alternata*, *Trichothecium roseum*, *Fusarium avenaceum*, *Cadophora malorum*, and *Neofabraea vagabunda*. Notably, these last two fungal species have been reported for the first time in Morocco as pathogens of stored apples. These data affirm that the high losses of apples in Morocco, attributed primarily to *P. expansum* and *B. cinerea*, pose a significant threat in terms of reduced production and diminished fruit quality. Hence, adopting controlled atmosphere storage chambers and implementing good practices before apple storage is crucial.

## 1. Introduction

The cultivation of apple trees in Morocco boasts a substantial annual production, reaching around 889,736 tons, with a harvested area spanning 52.550 ha [1]. Predominantly found in regions characterized by high and medium altitudes and featuring cold winters, apple tree cultivation is concentrated in key areas, notably Fez−Meknes (Meknes, Elhajeb, Ifrane, and Sefrou) and Draa−Tafilalet (Midelt and Zaïda). These two regions collectively account for 67% of all apple tree plantations in Morocco [2]. Harvesting occurs at the onset of ripening, primarily because a significant portion of the production is intended for storage in refrigeration stations. This strategic approach allows extended marketing periods, especially for fruit collected in large quantities over a very short period [3]. As a result, using contemporary preservation technology, apples can be stored for a duration ranging from 7 to 11 months [4]. However, during the storage period, apples are susceptible to various attacks from a spectrum of storage diseases, including those of physiological and fungal origin [5]. These attacks result in considerable losses, estimated at 20–25% annually, primarily attributed to fungal diseases [6]. 

Postharvest apple diseases are caused by several fungal pathogens, leading to significant economic losses [7]. The major losses are attributed to fungi belonging to two distinct groups, which differ in their methods of fruit contamination. The first group infects the fruit through wounds caused by weather accidents or mishandling during harvest. The second group enters the fruit via lenticels, which are often represented by slow-growing fungi with symptoms appearing during storage. Notably, diseases resulting from injuries pose a real threat to apple production [8]. The main postharvest fungal diseases affecting apples include blue mold caused by *Penicillium expansum*, gray rot caused by *Botrytis cinerea*, brown rot caused by *Monilinia* sp., rot caused by *Altenaria* sp., and rot caused by *Gloeosporium album* [9].

Blue mold, also known as soft or wet rot, is the most important postharvest apple disease [10]. Beyond causing visible rots on the affected fruit, *Penicillium* spp. is responsible for these rots, producing the mycotoxin patulin, which is considered hazardous to human health. Elevated patulin levels render the attacked fruit unsuitable for human consumption and processing [11]. These pathogens exhibit growth even at temperatures as low as −3 °C, and their conidia can germinate at 0 °C. Various sources contribute to the presence of this pathogen, including organic debris in the orchard soil, dead tree bark, as well as the air and walls of storage warehouses [12]. Gray mold caused by the necrotrophic pathogen *B. cinerea* is a widespread postharvest apple disease. This disease can cause significant losses on apples during storage, especially in untreated fruit. The disease mainly arises from infection of wounds, such as cracks in the stem bowl area of apple fruit and punctures and bruises that are created during fruit picking and postharvest handling. However, *B. cinerea* can also infect apples during the blooming stage or just after fruit set through the open calyx of the fruit, although the symptoms of the disease only appear on the infected fruit during conservation. Furthermore, the spread of gray mold occurs simply through contact between the rotten fruit and the surrounding healthy fruit during storage [13,14]. Alternaria rot is a prevalent apple fungal disease globally, although it rarely results in substantial commercial losses. An increase in disease incidence has been linked to the use of postharvest benzimidazole for blue mold and gray rot control [15]. Initial infections may occur in the orchard at the flowering stage or in storage facilities, with symptoms appearing on fruit within two months of cold storage. *Alternaria* sp. has been reported as a pre- and postharvest pathogen on apples, and its symptoms may be confused with damage caused by codling moths in some cases [16]. To highlight the incidence of the three aforementioned diseases, a previous study on postharvest rot of apples in Greece showed that the percentage of occurrence of blue mold, gray mold, and Alternaria rot was 44.2%, 23.6%, and 16.1%, respectively [17]. Brown rot, a common fruit rot with similar symptoms across hosts, is primarily caused by the fungi *Monilinia fructigena*, *M. laxa*, or *M. fruticola* [18]. However, researchers have considered brown rot on apples to be a minor disease with an incidence of 5.3% [17]. *M. fructigena* is a serious pathogen of stone fruit [19]. *M. fructigena* can infect flowers, immature and mature fruit, and small branches. Huge losses can occur in warm, humid, and rainy weather conditions that promote the development of diseases and the absence of fungicide treatment during flowering or just before ripening [12,20]. Additional losses are possible in storage conditions if the fruit is not treated properly during harvest. Systematic removal of mummified fruit and infected twigs from an orchard can significantly reduce the incidence of this disease [12,21]. 

Bull’s eye rot is a postharvest apple disease that causes significant economic damage [22]. Studies in Italy and Chile have shown that this disease appears on most apple cultivars, with an incidence ranging from 10% to 20%, and it can exceed 40% in years favorable to pathogen infection [23,24]. It is caused by various species of fungi belonging to the genus *Neofabraea* (*N. vagabunda*, *N. malicorticis*, *N. kienholzii*, and *N. perennans*), with *N. vagabunda* being a major contributor to apple bull’s eye rot [25,26]. Bull’s eye rot begins as a latent infection occurring in the orchard, with the pathogen living quietly in the fruit for several months after harvest (usually 2 to 3 months) before causing symptoms of disease [27].

To control these pathogens, the application of fungicides either prior to or directly after harvesting is a key strategy for the successful management of postharvest decays of apples during storage [5]. Furthermore, ensuring cleanliness in the storage environment and employing sanitized equipment proves to be an effective measure for minimizing apple infections within cold storage rooms [28]. Recognizing the rise of strains resistant to conventional active ingredients, biological control is increasingly adopted as a rational alternative to control postharvest fruit diseases [29].

To the best of our knowledge, the only study targeting the identification of fungal agents responsible for postharvest diseases of apples in Morocco was carried out by Attrassi et al. [5]. This study associated several fungal species, such as *P. expansum*, *A. alternata,* and *T. roseum*, with postharvest apple diseases. However, the identification relied solely on morphological traits, leading to an incomplete definitive characterization. Indeed, the pathogenicity of the isolates on apples was not assessed. Hence, there is notable interest in undertaking molecular studies to precisely characterize these fungal pathogens that cause apples to decay in Morocco and to assess their pathogenicity. Furthermore, there exists a significant gap in understanding apple storage conditions in Morocco. Thus, it is imperative to underscore the primary factors that influence apple losses in packinghouse and storage stations. To address these concerns, our study aims to (i) investigate the storage conditions of apples in refrigeration stations, (ii) identify apple storage diseases in four study regions, and (iii) assess the prevalence of these fungal diseases.

## 2. Results

Surveys using questionnaires were conducted in 46 apple storage stations in Morocco to highlight the apple storage conditions.

### 2.1. Storage Conditions for Apples in Storage Warehouses

**Figure 1:**
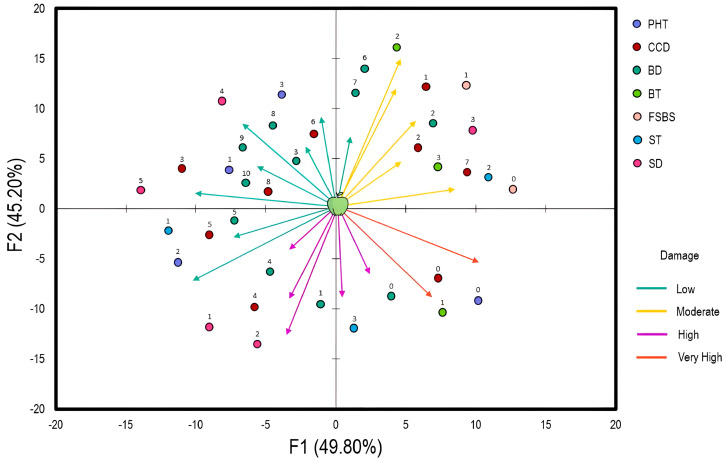
Multiple Component Analysis (MCA) highlighting the relationship between apple storage conditions and the observed damage (low = 110%; moderate =10−20%; high = 20−30%; very high = 30−40%). The arrow direction indicates the correlation between each variable and the correspondence axes (F1 and F2). The arrow length shows the relative contribution of the variables to the axes and storage conditions. The numbers above circles represent the attributes of each variable studied in Table 3. Abbreviations: PHT, postharvest treatment; CCD, cold chamber disinfection; BD, box disinfection; BT, box type; FSBS, fruit sorting before storage; ST, storage temperature; SD, storage duration.

To evaluate the relationship between apple storage conditions and perceived damage, a multiple-component analysis was established (Figure 1). The fraction of variances was 49.8% and 45.2%, respectively. The damage was categorized into four classes in terms of severity (low, moderate, high, very high). The analysis showed that postharvest treatment (PHT), cold chamber disinfection (CCD), and box disinfection (BD) are strongly related to the low damage observed in apples. PHT was based on Pelt 44 (Thiophanate-methyl: 1.5–2.5 g/L) applied as a dip treatment, Bavistin (Carbendazim: 0.5–1 g/L) applied as a spray treatment, and Score (Difenoconazole: 0.1–0.5 g/L) applied as a dip or spray treatment. These products were used prior to fruit sorting (FS) and before the appearance of any symptoms of fungal decay. On the other hand, detergents (quaternary ammonium compounds, chlorine-based cleaners, peroxyacetic acid), bleach, fumigation, and Pelt 44 (5 g/L as spray or fog treatment) were considered for CCD and BD. Regarding box types (BT), moderate damage was observed when wood and plastic boxes were used separately. The same result was obtained with fruit sorting before storage (FSBS). As for storage temperature (ST) and duration (SD), high temperatures increase the damage trend, whereas extensive durations reverse the trend.

**Figure 2:**
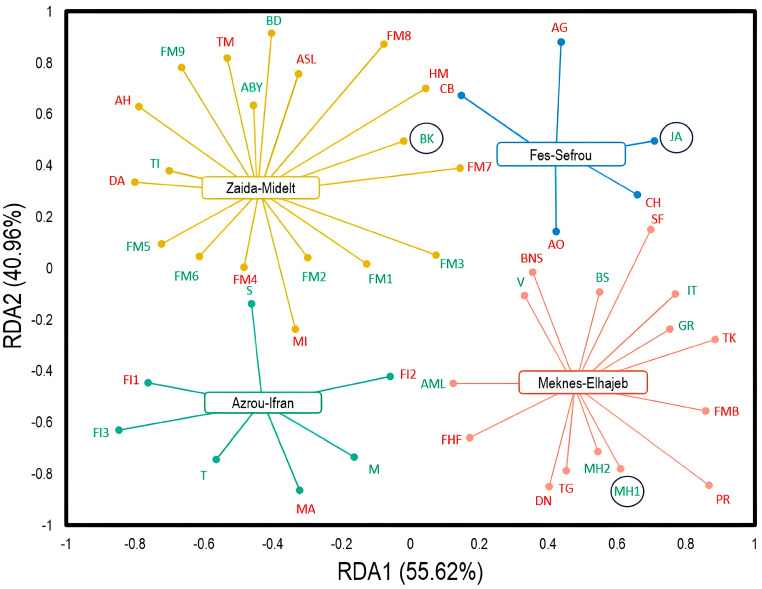
Redundancy analysis (RDA) showing the distribution trend of Moroccan apple conservation stations in terms of observed damage. The codes represent the stations studied. Red−colored stations represent severely damaged entities (high to very high damage = 20−40%). Green-colored stations represent entities with less damage (low to moderate damage = 1−20%). The circled entities represent the controlled atmosphere conservation stations.

The tendencies of the surveyed Moroccan apple storage warehouses in terms of observed damage were explored through redundancy analysis (RDA) (Figure 2). The fractions of variance for the associated axes (RDA1 and RDA2) were 55.62% and 40.96%, respectively. Large conservation stations with less damage (1–20%) were observed in the Zaida−Midelt and Azrou−Ifran regions. Conversely, the regions of Fez−Sefrou and Meknes−Elhajeb have conservation warehouses with a high damage percentage (40%). Interestingly, three conservation stations (JA, MH1, and BK) recorded the lowest damage percentages (1–10%), as they are controlled atmosphere storage warehouses.

In the following section, the fungal pathogens affecting the apples were identified morphologically, following macroscopic and microscopic descriptions.

### 2.2. Morphological Identification of Pathogens Causing Fungal Diseases in Postharvest Apples 

Based on morphological characteristics, the results indicated that postharvest fungal diseases affecting apples are caused by seven pathogens: *Penicillium expansum* (Aby4), *Botrytis cinerea* (PR1), *Alternaria alternata* (Ag4), *Trichothecium roseum* (AI3), *Fusarium avenaceum* (AML28), *Cadophora malorum* (PRL1), and *Neofabraea vagabunda* (MY2).

**Figure 3:**
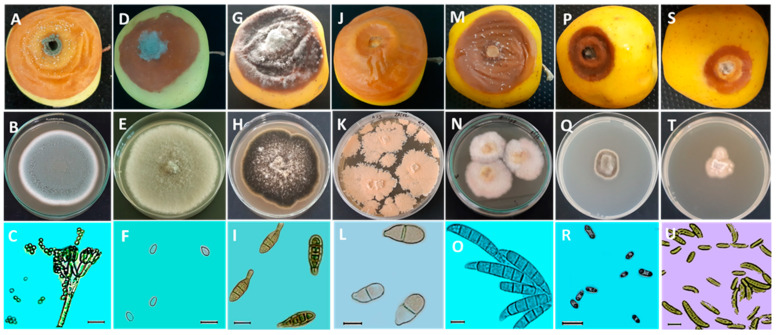
Morphological characteristics of the main diseases affecting apples during storage. (**A**,**D**,**G**,**J**,**M**,**P**,**S**): appearance of apple (Golden Delicious) rot after inoculation with spore solution (10^4^ spores/mL) and incubation for 14 days at 25 °C. (**B**,**E**,**H**,**K**,**N**,**Q**,**T**): pathogen colonies on Potato Dextrose Agar (PDA) medium after incubation for 7 to 14 days at 25 °C. (**C**,**F**,**I**,**L**,**O**,**R**,**U**): microscopic observation (×40) of conidia and mycelium of pathogens. A, B, and C: blue mold caused by *Penicillium expansum.* (**D**,**E**,**F**): gray mold caused by *Botrytis cinerea*. (**G**,**H**,**I**): Alternaria rot caused by *Alternaria alternata.* (**J**,**K**,**L**): bitter rot caused by *Trichothecium roseum.* (**M**,**N**,**O**): Fusarium rot caused by *Fusarium avenaceum*. (**P**,**Q**,**R**): side rot caused by *Cadophora malorum.* (**S**,**T**,**U**): gloeosporiosis caused by *Neofabraea vagabunda*. Scale bar = 10 μm.

The cultivation of *P. expansum* on the PDA medium resulted in fungal colonies with a blue-green color, abundant sporulation, and a white edge (Figure 3B). The conidiophore of this fungal species is terverticillate. Indeed, the stipe branches into secondary branches that further divide into metulae, and these also branch into phialides that bear small-sized conidia of about 3.42 ± 0.2 × 3.03 ± 0.27 µm in diameter (Table 1), with a subglobose to elliptical shape (Figure 3C). Furthermore, the appearance of *B. cinerea* colonies on the PDA medium was characterized by a gray color with abundant mycelium (Figure 3E). The conidia of *B. cinerea* are unicellular and oval-shaped, with dimensions of 6.88 ± 0.75 × 4.52 ± 0.43 µm (Table 1). Regarding the *A. alternata*, the fungal colonies on the culture medium (PDA) are dark brown with irregular light beige margins. Over time, cushions appear toward the center with a light beige to white color (Figure 3H). The conidia are multicellular, elliptical, and pear-shaped. Their color ranges from pale brown to olive brown, and they generally have two to three transverse septa and occasionally one longitudinal septum. Their ends are formed by a somewhat elongated narrowed part, but this can sometimes be absent (Figure 3I). The average dimensions of these conidia are 26.46 ± 5.51 × 9.22 ± 1.47 µm (Table 1). However, the colonies of *T. roseum* are flat and granular, with a yellowish-beige color (Figure 3K), and contain bicellular conidia that are elliptical to pear-shaped with a slanted basal termination (Figure 3L). They measure 20.69 ± 2.24 µm in length and 9.21 ± 0.94 µm in width (Table 1). The *F. avenaceum* produces colonies with dense aerial mycelium, initially white and later changing from yellow to pink (Figure 3N). The macroconidia of this isolate are falcate, slightly curved, generally exhibiting five septa, and have elongated apical and basal cells (Figure 3O). The average dimensions of the conidia are 47.87 ± 5.81 × 6.96 ± 0.59 µm (Table 1). Nevertheless, the colonies of *C. malorum* displayed slow-growth mycelium with a fuzzy texture, a brown color, and a distinct beige margin (Figure 3Q). The conidia of PRL1 are unicellularalong to cylindrical, biguttulate, and have rounded ends (Figure 3R). These conidia have an average length of 6.46 ± 1.57 µm, and their average width is 2.97 ± 0.62 µm (Table 1). In contrast, the fungal pathogen *N. vagabunda* is characterized by slow-growing colonies that are raised in the center, with a whitish to pale beige color and an irregular contour (Figure 3T). The conidia are unicellular, aseptate, fusiform, and occasionally curved at the ends (Figure 3U). Their average dimensions are 17.05 ± 4.5 × 3.51 ± 0.53 µm (Table 1).

In the following section, the pathogens of apples were identified using a molecular approach, starting with DNA extraction, followed by PCR, and concluding with sequencing of a DNA region.

### 2.3. Molecular Identification and Phylogenetic Analysis

The fungal species isolates identified on the basis of their morphological characteristics were confirmed through molecular sequencing of the internal transcribed spacer (ITS) region of rDNA using the ITS1 and ITS4 primers. Indeed, isolate “Aby4” was identified as *Penicillium expansum* with accession number (AN) OR426630, “PR1” was identified as *Botrytis cinerea* under AN OQ691642, “Ag4” was *Alternaria alternata* with AN OQ691639, “AI3” was identified as *Trichothecium roseum* submitted under AN ON680682, “AML8” was *Fusarium avenaceum* with AN OR426633, “PRL1” was *Cadophora malorum* under AN OR426632, and finally, isolate “MY2” was *Neofabraea vagabunda* submitted under AN OR426631 (Table 2). In addition, *P. expansum* and *N. vagabunda* were also identified by sequencing primer pairs Bt2a and Bt2b of the β-tubulin gene region with AN OL802926 and AN OR753458, respectively (Table S1), while *A. alternata* was confirmed by specific primer pairs AMT4-EMR-F and AMT4-EMR-R (Table S2).

**Figure 4:**
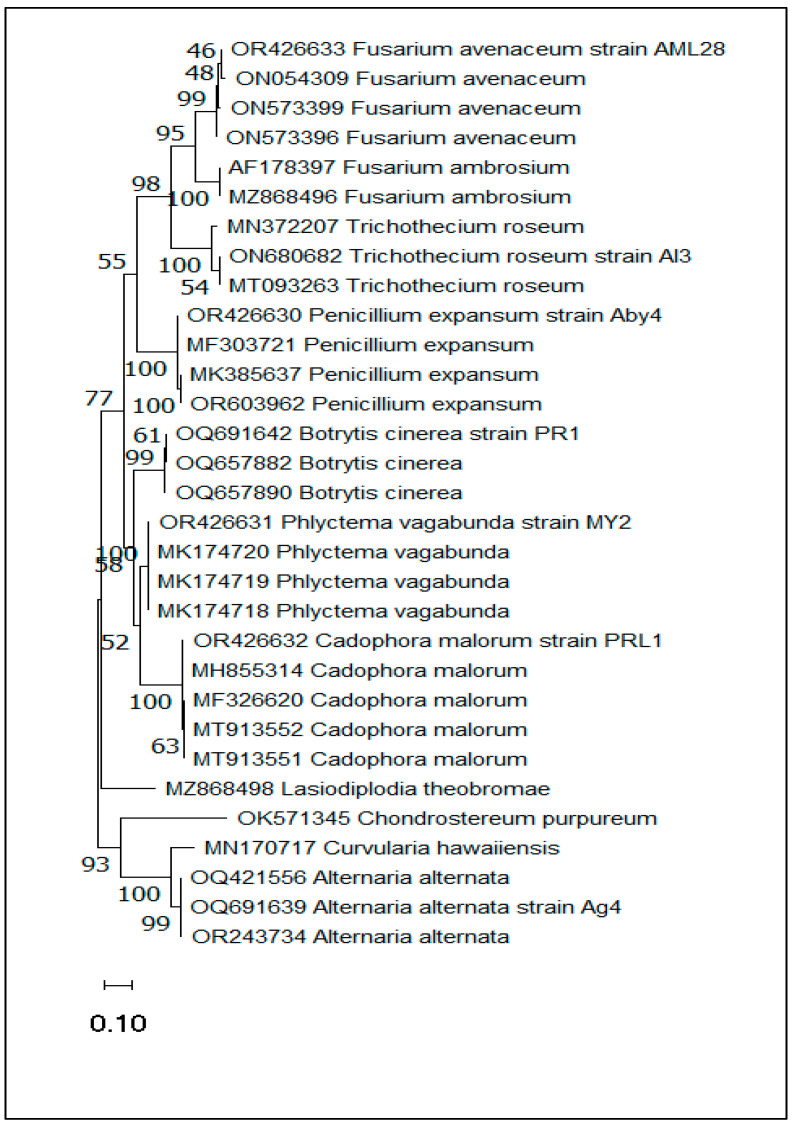
Phylogenetic tree generated in MEGA11 software (version 11.0.8 build 210914) using a Kimura two-parameter model based on maximum likelihood analysis of nucleotide sequences of the ITS gene of the main pathogens affecting apple on storage.

The phylogenetic tree was constructed by MEGA11 software (version 11.0.8 build 210914) using the maximum likelihood method and the two-parameter Kimura model. The clustering percentage of associated taxa is indicated next to the branches. The tree is represented to scale, with branch lengths measured in the number of substitutions per site. This analysis covered 31 nucleotide sequences, including the seven species reported in the present study, where each strain was grouped with its high support value reference. Accordingly, this phylogenetic tree showed high similarity between these seven species and the reference species (Figure 4).

### 2.4. Symptoms of Different Pathogens Isolated from Apples during Storage 

In the paragraph below, the pathogens isolated from the collected apples were tested to assess their pathogenicity and describe their symptoms.

Inoculation of pathogenic fungi on healthy cv. Golden Delicious apples resulted in rots with a different appearance. Specifically, the “Aby4” isolate caused rot in a circular form with a light brown color on the outside and inside, with distinct edges. Mold develops on the surface of the rot, initially white and then bluish-green (Figure 3A). However, rot caused by “PR1” is characterized by a distinct and irregular outline and a brown color. The decay’s center became densely covered with a gray fuzz (Figure 3D). On the other hand, the fungal isolate “Ag4” initially causes firm black necrosis, which later evolved into a soft, brown texture. The surface of the decay is covered with a typical ashy gray fuzz (Figure 3G). Meanwhile, the “AI3” isolate caused an alteration with a nearly irregular outline, a soft texture, and a light brown color. The characteristic bitter taste of the healthy part of the flesh was observed (Figure 3J). Symptoms caused by the pathogenic isolate “AML28” manifested as a variable-sized wet brown rot with pink cushions on the decay’s surface (Figure 3M). Regarding the “PRL1” isolate, the induced rot was characterized by a small diameter, with a dark brown color toward the tip and a light brown color toward the center (Figure 3P). Lastly, the “MY2” isolate resulted in a rot with a small diameter, exhibiting a light brown color toward the edges and beige toward the center. The latter was white (Figure 3S).

### 2.5. Pathogenicity of the Isolates

In the upcoming paragraph, the virulence of the apple diseases detected in this study was compared based on the diameter of the rot on the apples.

The results of the pathogenicity tests showed that all seven fungal isolates were pathogenic, as they demonstrated the ability to induce lesions into wounds on healthy apples upon inoculation. The obtained symptoms were as previously described in Section 2.2 (Figure 3). However, the control did not display any lesions on the apple surface. The fungal isolates were re-isolated from the decayed apples, and identification was performed following Koch’s postulates. This confirmed that *P. expansum* caused blue mold rot in apples, *Botrytis cinerea* led to gray mold rot, *A. alternata* induced alternariosis, *T. roseum* was responsible for bitter rot, *F. avenaceum* was the causal agent of Fusarium rot, *C. malorum* resulted in Cadophora rot, and *N. vagabunda* caused apple gloeosporiosis.

**Figure 5:**
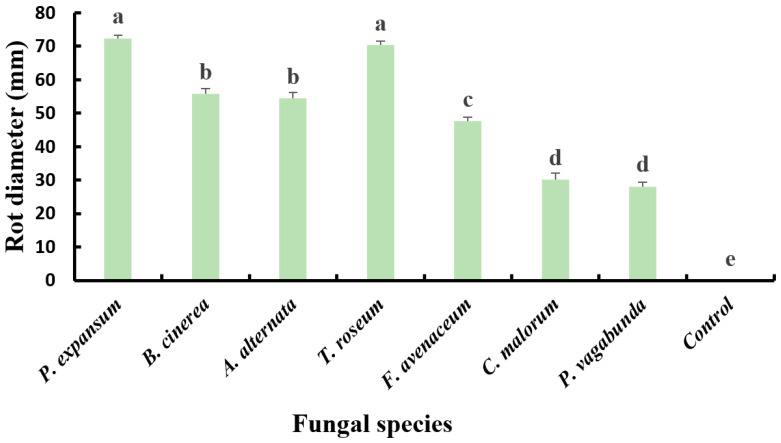
Diameter of apple (cv. Golden Delicious) rots inoculated with spore solution (1 × 10^4^ spores/mL) and sterile distilled water (control) after incubation for 14 days at 25 °C. Diameters with the same letter are not significantly different according to Duncan test (*p* < 0.05).

Statistical analysis of apple lesion diameters, inoculated with 50 µL of spore suspension (10^4^ spores/mL) and incubated in plastic boxes at 25 °C for 14 days in growth chambers, revealed a significant difference (*p* < 0.05) in the pathogenicity of the seven fungal isolates. The most virulent isolates were *P. expansum* and *T. roseum*, with lesion diameters of 72.39 ± 0.83 mm and 70.29 ± 1.18 mm, respectively. Next were *B. cinerea* and *A. alternata*, which caused substantial lesions with diameters of 55.74 ± 1.58 mm and 54.45 ± 1.83 mm, respectively. *F. avenaceum* was moderately virulent, resulting in a rot with a diameter of 47.56 ± 1.31 mm. On the other hand, *N. vagabunda* and *C. malorum* are the least aggressive isolates, inducing smaller lesions with diameters of approximately 28.09 ± 1.34 mm and 30.18 ± 1.86 mm, respectively (Figure 5).

### 2.6. Prevalence of Fungal Pathogens Affecting Postharvest Apples

In the next section, the prevalence of each pathogen was determined based on the morphological identification of 190 fungal isolates from apples.

Sampling symptomatic apples collected from 46 storage stations in the Fez−Meknes and Draa−Tafilalet regions over 3 years resulted in the isolation of 190 fungal isolates. Their virulence capacity was revealed through pathogenicity testing. Based on their morphological characteristics, *P. expansum* was the most prevalent among the obtained pathogenic isolates (137 isolates), accounting for 72.1% of the total number of fungal species causing postharvest apple diseases. Next, *B. cinerea* constituted 18.42% of all isolated pathogens (35 isolates). Other species (*T. roseum*, *F. avenaceum*, *C. malorum*, and *N. vagabunda*) had a low representation, with a percentage of 2.1%.

## 3. Discussion

The majority of storage facilities in the study area primarily use ordinary cold chambers (93%), whereas a smaller percentage (7%) adopt controlled atmosphere chambers. In the context of controlled atmosphere (CA) storage, this method involves maintaining oxygen and carbon dioxide concentrations at approximately 1–5% for each gas, a departure from the oxygen-rich (around 21%) and low carbon dioxide (close to 0.03%) levels found in normal ambient air. The intentional adjustment to lower oxygen levels and increased carbon dioxide levels in CA storage slows down the ripening process, impedes the development of certain storage disorders, and decelerates the growth of postharvest fungal diseases. These effects collectively contribute to extending the shelf life of apples and preserving their quality when compared with standard cold storage rooms [30]. The data collected in this study indicate that the storage temperature ranges from 0 °C to 4 °C, depending on the apple variety. These values are suitable for maintaining the quality of various apple varieties during storage [31].

Based on the conducted surveys, three active substances are commonly used for postharvest treatment in cold storage facilities in the Fez−Meknes and Draa−Tafilalet regions. These substances include thiophanate-methyl and carbendazim from the benzimidazole family, as well as difenoconazole, which belongs to the demethylation inhibitor group. Benzimidazole fungicides exert their mode of action by targeting the microtubules of fungal cells, which are essential components of the fungal cytoskeleton that play a crucial role in various cellular processes, including cell division, intracellular transport, and maintenance of cellular shape [32]. However, resistance to benzimidazole fungicides has been detected in numerous fungal species and is correlated with point mutations in the β-tubulin gene, leading to amino acid sequence alterations at the benzimidazole binding site [33]. Demethylation inhibitors (DMI) exert their mode of action by inhibiting the activity of an enzyme called 14-α-demethylase, encoded by the CYP51 gene in fungi. This enzyme plays a crucial role in the biosynthesis of sterols, which are among the main constituents of the fungal cell membrane [34]. The main mechanisms of resistance to DMI involve mutation of the CYP51 14α-demethylase gene [35] or overexpression of this gene [36]. In this context, Malandrakis et al. [37] demonstrated that isolates of *P. expansum* were resistant to benzimidazole, and Sholberg et al. [38] also reported that *P. expansum* developed resistance to benzimidazole fungicides. On the other hand, a previous study conducted by Jurick et al. [39] highlighted that the product Academy, based on difenoconazole, exhibited both curative and protective activities in controlling *Penicillium* spp. populations, the causal agents of blue mold in stored apples.

These results revealed that several factors affect the percentage of apple losses during storage. Indeed, the postharvest treatment of apples and their sorting before storage in well-disinfected premises using correctly disinfected plastic boxes considerably reduced the percentage of apple losses in storage facilities, whereas the opposite was observed under less controlled conditions. Abi Tarabay et al. [40] proved that proper postharvest practices significantly reduce the percentage of losses and maintain the quality of apples. The estimated values of apple production losses in the surveyed refrigeration stations reached up to 40% in some storage warehouses. In Yamen, losses caused by fungal diseases are estimated at 20–25% [6]. According to Spadaro and Droby [41], fruit losses in Europe, North America, and Oceania can escalate to 29%, while in Asia, Africa, and Latin America, the impact is even more pronounced, with losses reaching up to 38%.

The results obtained from morphological and molecular characterization have demonstrated a diversity of fungal species causing postharvest apple diseases in Morocco, namely *P. expansum*, *B. cinerea*, *A. alternata*, *T. roseum*, *F. avenaceum*, *C. malorum*, and *N. vagabunda*. The morphological traits of these isolates are consistent with descriptions in several studies [42,43,44,45,46,47,48]. Pathogenicity tests and Koch’s postulates revealed that these fungal species are pathogenic to apples and induce blue mold, gray mold, alternariosis, bitter rot, Fusarium rot, Cadophora rot, and gloeosporiosis, respectively. It is important to mention that *N. vagabunda* (anamorph *Phlyctema vagabunda*) causes the postharvest disease known as gloeosporosis, also referred to as apple bull’s eye rot [24].

Comparing our study with previous studies, Wenneker and Köhl [49] isolated *Neofabraea* spp., *Botrytis* spp., *Penicillium* spp., *Fusarium* spp., *Alternaria* spp., and *Cladosporium* spp. from decayed apples collected from packing houses in different regions of the Netherlands. Furthermore, a recent study in Poland by Głos et al. [50] highlighted the emergence of several postharvest apple diseases, including blue mold (*P. expansum*), gray mold (*B. cinerea*), bull’s eye rot (*Neofabraea* spp.), brown rot (*Monilinia* spp.), alternariosis (*Alternaria* spp.), and new apple storage diseases caused by *Colletotrichum* spp., *Neonectria ditissima*, and *Diaporthe eres*. In addition, Dai et al. [51] demonstrated that *T. roseum* is a major pathogen of apples in China, and Spadaro et al. [52] reported that *C. malorum* is a pathogenic agent causing apple side rot. 

The analysis of the pathogenicity results of the seven fungal species confirmed that the most virulent isolate was *P. expansum*, inducing blue mold with the largest diameter. Consistent with other studies, *P. expansum* has been identified as the most aggressive fungal pathogen affecting stored apples [53,54,55]. The high virulence of *P. expansum* on apples can be attributed to several key factors. Cell wall-degrading enzymes (CWDEs), particularly polygalacturonases, play an essential role in promoting tissue maceration and pathogen colonization. These CWDEs significantly contribute to the virulence of *P. expansum* on apples [56,57]. Another strategy employed by *P. expansum* to enhance its virulence is to acidify the host tissues. By producing gluconic, citric, and fumaric acids, the fungus lowers the pH of the host, thereby promoting the optimal activity of CWDE [58,59]. In addition to these mechanisms, *P. expansum* isolates produce various secondary metabolites, some of which, such as patulin and citrinin, cause cellular damage. These potential mycotoxins contribute to virulence by accentuating the pathogenic effects of *P. expansum* on apples [60,61]. On the other hand, among the seven isolates obtained, the least virulent was *N. vagabunda*, the causal agent of apple gloeosporiosis (bull’s eye rot). The low pathogenicity of this fungal species could be explained by the fact that it is a latent pathogen that only becomes obvious after several months of apple storage in storage facilities. This reasoning has been confirmed by Wenneker and Thomma [62]. In this context, Cameldi et al. [24] stated that bull’s eye rot is a latent infection that initiates in the orchard, but the pathogen remains dormant within the fruit for several months after harvest before triggering disease symptoms. 

Evaluation of the prevalence of fungal pathogens isolated from rotten apples in different storage stations over a 3-year sampling period revealed a significant dominance of *P. expansum*, which is responsible for blue mold (72.1%). Consistent with these findings, Rharmitt et al. [11] reported that 79.5% of the pathogenic fungi belonged to the genus *Penicillium* spp. Furthermore, Amiri and Bompeix [28] demonstrated the substantial contribution of these species to apple losses during storage in France, with similar results noted by Konstantinou et al. [17] in Greece. Another study also highlighted *P. expansum* as the most widespread and economically important postharvest pathogen responsible for apple rot [63]. Similarly, Vico et al. [44] stated the potential for blue mold to lead to significant economic losses during storage.

The heightened incidence of postharvest fungal disease in apples may be attributed to the ability of these pathogens to spread through direct contact between healthy and contaminated fruit, resulting in a 15–20-fold multiplication of the initially infected fruit [64]. The incidence of *B. cinerea* ranks second among apple pathogens, constituting 18.42% of all isolates obtained. Notably, the combined incidence of blue mold and gray mold accounts for most detected rots (90.52%). This result aligns with findings from several other research works, such as those of Konstantinou et al. [17], who identified *P. expansum* and *B. cinerea* as the predominant pathogens. Another study also confirmed that the most commonly isolated pathogens from decayed apples were *Penicillium* spp. and *B. cinerea* [65]. Similarly, Jijakli and Lepoivre [66] declared that blue mold and gray mold are considered to be the major postharvest diseases of apples.

Apple production is a sector of great economic importance in Morocco. However, postharvest diseases leading to significant losses represent a major challenge. Thus, broadening our understanding of the diversity of species causing these diseases, their pathogenicity, and the key factors contributing to these losses appears essential to minimize the damage. To the best of our knowledge, this is the first investigation in Morocco addressing the molecular characterization of the pathogens responsible for these diseases, along with exploring the correlation between losses and key factors in storage conditions. The particularity of this investigation lies in the fact that its results constitute valuable data that could assist managers in designing appropriate management strategies during storage aimed at minimizing apple production losses.

## 4. Materials and Methods

### 4.1. Study Area and Sampling

**Figure 6:**
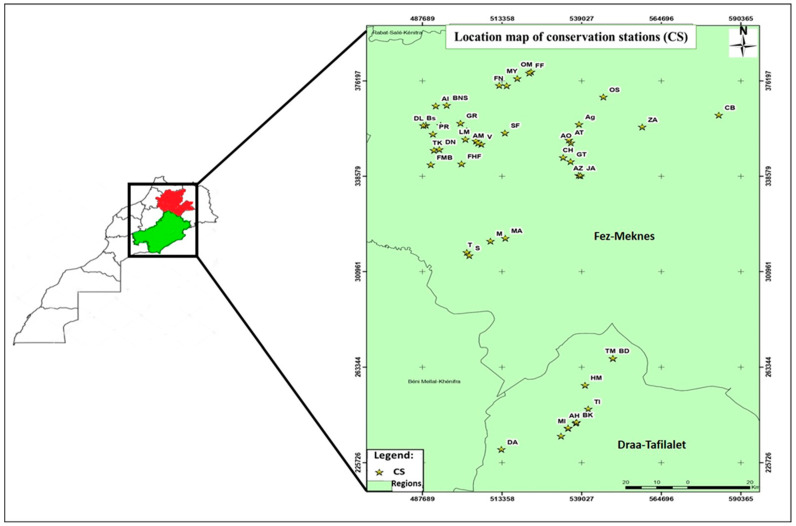
Map of Morocco showing the location of apple conservation stations where sampling was performed in the two regions of Fez−Meknes and Draa−Tafilalet, prepared using ArcGIS 10.3.1 software.

To ensure a comprehensive and representative sampling, rotten apples showing different symptoms were systematically collected from the cold chambers of 46 packing stations in most of Morocco’s apple-producing regions (Figure 6). This sampling initiative spanned three seasons (2019–2020, 2020–2021, and 2021–2022). All collected samples were carefully placed in appropriately labeled plastic bags and subsequently transported to the phytopathology laboratory at the National School of Agriculture in Meknes. In tandem with the sample collection process, surveys employing questionnaires were conducted at all 46 apple storage stations in Morocco (sample size: *n* = 46), specifically in the regions under scrutiny for this study. These surveys aimed to elucidate the prevailing storage conditions for apples in refrigeration stations, namely, postharvest treatment (PHT), cold chamber disinfection (CCD), box disinfection (BD), box type (BT), fruit sorting before storage (FSBS), temperature (ST), and storage duration (SD) (Table 3). These conditions can provide a valuable context for the comprehensive assessment of storage diseases and contributing factors. In addition, the damage severity (%) targeting apple fruit was accordingly apprehended from the surveys and categorized into four distinct classes in terms of rotting symptoms caused by fungal pathogens: low (1–10%), moderate (10–20%), high (20–30%), and very high (30–40%).

### 4.2. Isolation and Purification of Pathogens

The collected samples underwent a thorough process to ensure proper handling and analysis. First, the samples were washed with running water to remove external contaminants. Then, they were disinfected using a 2% sodium hypochlorite solution, followed by two rinses with sterile distilled water. The samples were then air-dried in a laminar flow hood. Using a sterile scalpel, three pieces were carefully excised from the front of the rot on each sample. These segments were placed in Petri dishes containing Potato Dextrose Agar (PDA) culture medium. The prepared dishes were then incubated at 25 °C for seven days in darkness using an IN 30 cultivator (Memmert GmbH Co., Koln, Germany). To obtain pure isolates, several subcultures on PDA medium were performed. The fungi obtained through this process were stored at 4 °C until use [17,49].

### 4.3. Pathogenicity Test

To test the pathogenicity of the pure isolates obtained, spore suspensions were prepared by adding 10 mL of sterile distilled water (SDW) to each 10-day-old fungal culture. To separate spores from mycelium and agar debris, the suspension was filtered using Whatman paper (N°1). The final concentration was adjusted to 1 × 10^4^ spores/mL using a hematocytometer. For this test, healthy apple fruit (*Malus domestica* cv. Golden Delicious) of the same size and showing no visible injury or rot were harvested at the maturity stage and did not receive any postharvest treatment. The fruit was disinfected as described in Section 4.2. They were wounded in the equatorial part in three equidistant sites (3 mm in diameter and 4 mm in depth) using a sterile stainless steel rod. Each wound was inoculated with 50 μL of each spore suspension (1 × 10^4^ spores/mL Apples inoculated with sterile distilled water were used as controls [67]. The fruit was then placed in sterile plastic boxes and incubated in a culture chamber for 14 days at 25 °C. The diameter of each fruit was measured using a caliper, and a re-isolation on the PDA medium was performed according to Koch’s postulate [47]. The most virulent isolate among others causing the same disease was chosen for a detailed description of the symptoms on apples, as well as for comparing the pathogenicity among all identified fungal diseases. Regardless of the pathogen, all experiments were repeated twice over time with 3 replicates. 

### 4.4. Morphological Identification

Morphological identification of fungal species was performed using determination keys [8,68,69,70,71,72]. The morphological characteristics observed are mainly the color and shape of the pure fungal colonies obtained after 7 to 14 days of incubation at 25 °C and microscopic observations of the mycelium and conidia [73,74]. The dimensions of conidia were measured for 30 conidia for each isolate as described by Díaz et al. [75] using a light microscope BX51 (Olympus, Tokyo, Japan) equipped with a camera (Olympus C-5060 associated with Touch-Scope Integrated powerful software, 3.7).

### 4.5. DNA Extraction, PCR Amplification, and Molecular Identification

The extraction of genomic DNA was performed by adopting the extraction method described by Doyle and Doyle [76]. Indeed, the equivalent of one square centimeter of each sample was taken and placed in an extraction tube. Then, 500 µL of the extraction buffer was added. The mixture was crushed using a pestle, vortexed, and incubated for 30 min at 65 °C in a water bath. During incubation, the tubes were mixed by rocking. Afterward, centrifugation was performed at 13,000 rpm for 5 min. About 400 μL of the supernatant was recovered, and an equivalent quantity (400 μL) of chloroform/isoamylalcohol (24/1) was added. A slight agitation for 5 min and centrifugation at 14,000 rpm for 5 min were performed. A volume of 350 µL of the supernatant was collected and precipitated with 350 µL of isopropanol. The tubes were rocked to be mixed, and another centrifugation at 14,000 rpm for 10 min was performed. The supernatant was discarded, and 500 µL of 70% ethanol was added to the pellet. After being vortexed in the tubes, they were centrifuged for 5 min at 14,000 rpm. The supernatant was discarded, and the pellet was dried in an oven at 60 °C (30 to 45 min) and taken up in 50 µL of sterile distilled water. Finally, the DNA thus obtained is stored at −20 °C. DNA quantification and quality assessment were performed using a NanoDrop (Jenway Genova Nano, Serial No 67281, Cole−Parmer Ltd stone, United Kingdom).

Polymerase chain reaction (PCR) of the internal transcribed spacer (ITS) region was performed using universal primers ITS1: TCCGTAGGTGAACCTGCGG and ITS4: TCCTCCGCTTATTGATATGC [77]. A final volume of 25 μL was used for each PCR reaction. The reaction mix included 5 µL of PCR buffer (5×), 1 µL (10 µM) of each primer, 0.2 µL (5 U/µL) of EnzimaGoTaq DNA polymerase (Bioline, London, UK), 15.3 µL of sterile distilled water, and 2.5 µL of genomic DNA. For the negative control, genomic DNA was replaced with SDW. PCR was performed using a thermocycler according to the following steps: first, an initial denaturation step for 3 min at 95 °C, followed by 35 cycles for denaturation for 35 s at 95 °C, annealing for 60 s at 55 °C, elongation for 2 min at 72 °C, and finally a final elongation for 10 min at 72 °C [78]. To confirm molecular identification, β-tubulin and AMT4-EMR primers were used (Table S2). Amplified PCR products were visualized on a 1.5% agarose gel (BIOLINE: Agarose, Molecular Grade) using a UV transilluminator (QUANTUM CX5 Edge—Gel Documentation System, Collegien, France) to evaluate the presence and size of amplicons after electrophoresis using Tris–borate–EDTA (TBE) buffer (×0.5) containing 5.39 g Tris (Sigma Life Science, Saint Louis in USA and Toronto in Canada), 2.75 g boric acid (Fisher Scientific International Company, Portsmouth, NH, USA), and 0.29 g EDTA (Ploysciences, Inc., Warrington, PA, USA) per 1 L of distilled water. The obtained PCR products were then sequenced using the Sanger method. ITS sequences were edited and aligned using DNAMAN^®^ software (version 6.0, Lynnon Biosoft, Quebec, Canada). The sequences obtained were checked using Blast search to identify similar sequences in the National Center for Biotechnology Information (NCBI) databases and then deposited in GenBank under unique accession numbers for each isolate. Phylogenetic analysis was performed using obtained ITS sequences to generate a phylogenetic tree using MEGA11 software (version 11.0.8 build 210914). The maximum likelihood method was adopted to estimate phylogenetic relationships. The support of each branch of the inferred tree was assessed using 1000 bootstrap replications.

### 4.6. Assessment of the Prevalence of Fungal Pathogens Affecting Postharvest Apples

The prevalence of fungal pathogens affecting postharvest apples was assessed based on morphological identification using the previously mentioned identification keys. The isolates were obtained from various symptomatic apples collected from the 46 storage stations in the Fez−Meknes and Draa−Tafilalet regions.

### 4.7. Statistical Analysis

The obtained data are presented as mean ± standard deviation (SD). For each experiment, analysis of variance (ANOVA) was conducted using IBM SPSS Statistics software (version 25). When the effect was significant for the pathogenicity test, Duncan’s test was applied to separate the means at *p* < 0.05. Redundancy analysis (RDA) was performed using the *skbio.math.stats.ordination* module integrated into Python libraries to showcase the tendencies of surveyed Moroccan apple fridges in terms of observed damage. Furthermore, Multiple Component Analysis (MCA) was employed using the *prince* module in Python (version 3.11) to depict the relationship between apple storage conditions and the observed damage.

## 5. Conclusions

Building upon the findings of this study, it is evident that apples stored in Morocco face a range of fungal diseases, with blue mold and gray rot being the predominant challenges. Addressing these issues requires a multifaceted approach. First, the implementation of postharvest treatments for apples has demonstrated efficacy in combating fungal diseases. These treatments play a crucial role in minimizing the impact of pathogens and enhancing the overall quality of stored apples. Moreover, maintaining a high level of cleanliness in storage environments and equipment is another key strategy. The reduction of potential sources of contamination and the implementation of rigorous hygiene practices contribute significantly to the prevention and control of fungal infections. This approach not only safeguards the apples but also supports the overall hygiene and quality standards of the storage facilities. In addition to postharvest treatments and cleanliness measures, this study highlights the noteworthy effectiveness of using controlled atmosphere rooms for apple preservation. This technology, which regulates oxygen and carbon dioxide concentrations, proves instrumental in extending the shelf life of apples while preserving their quality. Controlled atmosphere rooms create an environment that impedes the development of storage disorders and slows down the growth of postharvest fungal diseases, offering a comprehensive solution for prolonged apple storage. A holistic strategy involving postharvest treatments, strict cleanliness protocols, and the integration of controlled atmosphere rooms emerges as a robust approach to tackle the array of fungal diseases affecting stored apples in Morocco. Implementing these measures not only addresses current challenges but also sets the stage for enhancing the overall efficiency and sustainability of apple storage practices.

## Figures and Tables

**Table 1:** Conidia characteristics of the main pathogens affecting apples during storage.

		Conidia Length (μm)			Conidia Width (μm)			
Species	Isolate	Min	Max	Mean ± SD	Min	Max	Mean ± SD	Ratio (Length/Width)
***P. expansum***	Aby4	3.14	3.9	3.42 ± 0.2 d	2.56	3.53	3.03 ± 0.27 bc	1.13
***B. cinerea***	PR1	5.84	9.13	6.88 ± 0.75 c	4	5.84	4.52 ± 0.43 b	1.52
***A. alternata***	Ag4	16.73	38.88	26.46 ± 5.51 b	6.4	13	9.22 ± 1.47 a	2.87
***T. roseum***	AI3	17.36	24.36	20.69 ± 2.24 b	7.25	10.92	9.21 ± 0.94 a	2.25
***F. avenaceum***	AML28	40.61	64.51	47.87 ± 5.81 a	5.93	7.84	6.96 ± 0.59 ab	6.88
***C. malorum***	PRL1	4.18	9.07	6.46 ± 1.57 c	1.62	3.71	2.97 ± 0.62 c	2.17
***N. vagabunda***	MY2	11.78	30.36	17.05 ± 4.5 bc	2.09	3.89	3.51 ± 0.53 bc	4.86

Letters (a, ab, b...) following the mean ± SD represent homogeneous groups according to the Duncan post hoc test at *p* < 0.05.

**Table 2:** Detailed information on fungal isolates that cause postharvest apple diseases.

Isolate Code	Species	Sampling Year	Origin	GPS Coordinates	Accession Number	Query Cover	Similarity Percentage
**PRL1**	*Cadophora malorum*	2022	El Hajeb	N 33°47′43,5336″ W 5°29′41,226″	OR426632	99%	100% (MF326620)
**AML28**	*Fusarium avenaceum*	2022	El Hajeb	N 33°48′28,6812″ W 5°22′35,2884″	OR426633	99%	100% (ON573396)
**MY2**	*Neofabraea vagabunda*	2022	Meknes	N 33°59′ 36,8088″ W 5°12′2,4948″	OR426631	99%	100% (MK174720)
**Aby4**	*Penicillium expansum*	2021	Midelt	N 32°45′2,2896″ W 5°1′45,1776″	OR426630	99%	100% (MF303721)
**PR1**	*Botrytis* *cinerea*	2021	El Hajeb	N 33°47′43,5336″ W 5°29′41,226″	OQ691642	100%	100% (MN088689)
**Ag4**	*Alternaria alternata*	2021	Sefrou	N 33°49′46,5456″ W 4°59′7,5876″	OQ691639	99%	100% (MW509980)
**AI3**	*Trichothecium roseum*	2020	Meknes	N 33°53′44,61″ W 5°29′6,8028″	ON680682	99%	100% (MT093263)

**Table 3:** Environmental and storage conditions of the sampled apple conservation stations within the Fez−Meknes and Draa−Tafilalet regions.

**Region**	**CC**	**ST (°C)**	**RH (%)**	**CCT**	**CCD**	**AC**	**PHT**	**BT**	**BD**	**SD (Months)**	**FSBS**	**RY**
Fez−Sefrou	JA	0–1	90–95	CA (O_2_: 2–3%, CO_2_: 1.5–3%)	Bleach	GD	Pelt 44	Plastic	Bleach	10	Yes	2021
	AO	1.1–2.5	81–89	Normal	Detergent + Pelt44	GD	None	Wood and Plastic	Detergent + Pelt44	6	No	2021
	CH	1.1–2.5	81–89	Normal	Bleach + Pelt44	GD	None	Wood and Plastic	Bleach + Pelt44	7	No	2021
	Ag	2.6–4	70–80	Normal	Detergent	GD	None	Wood and Plastic	Detergent	6	No	2021
	CB	1.1–2.5	81–89	Normal	Detergent	GD	None	Wood and Plastic	Detergent	6	No	2021
Meknes−Elhajeb	IT	1.1–2.5	90–95	Normal	Pelt44 + Fumigation	GD	Pelt 44	Wood and Plastic	Bleach + Soda	9	Yes	2022
	BNS	2.6–4	81–89	Normal	Detergent	GD	None	Wood and Plastic	Detergent	6	No	2022
	FMB	1.1–2.5	81–89	Normal	None	GD	None	Wood	None	6	No	2022
	TK	1.1–2.5	81–89	Normal	Bleach	GD	None	Wood and Plastic	Bleach	7	No	2022
	Bs	0–1	90–95	Normal	Detergent + Pelt44	GD	Pelt 44	Plastic	Detergent + Pelt44	9	Yes	2022
	V	0–1	90–95	Normal	Pelt44	GD	Pelt 44 + Bavistin	Plastic	Pelt 44	10	Yes	2022
	Tg	1.1–2.5	81–89	Normal	Bleach	GD	None	Plastic	Bleach	8	No	2022
	DN	1.1–2.5	81–89	Normal	Detergent	GD	None	Wood and Plastic	Detergent	6	No	2022
	FHF	1.1–2.5	81–89	Normal	Detergent	GD	None	Wood and Plastic	Detergent	8	No	2022
	GR	0–1	90–95	Normal	Pelt44	GD	Pelt 44	Plastic	Pelt44	9	No	2022
	AML	0–1	90–95	Normal	Detergent	GD	Pelt 44	Plastic	Pelt44	10	Yes	2022
	PR	1.1–2.5	81–89	Normal	Pelt44	GD/StD/Fuji	None	Plastic	Detergent	7	No	2022
	MH1	1.1–2.5	90–95	CA (O_2_: 2–3%, CO_2_: 2–3%)	Bleach	GD	Score	Plastic	Bleach	9	No	2022
	MH2	0–1	90–95	Normal	Detergent + Pelt44	GD	Pelt 44 + Bavistin	Plastic	Detergent + Pelt44	6	No	2022
	SF	1.1–2.5	81–89	Normal	Bleach + VIROCID	Anna	Pelt 44	Wood and Plastic	Bleach + VIROCID	6	No	2022
Azrou−Ifran	M	1.1–2.5	81–89	Normal	Pelt44	GD	None	Wood and Plastic	Detergent	6	No	2020
	T	1.1–2.5	81–89	Normal	Pelt44	GD	None	Wood and Plastic	Pelt44	6	Yes	2020
	MA	1.1–2.5	81–89	Normal	None	GD	None	Wood	None	6	No	2020
	FI1	1.1–2.5	81–89	Normal	Detergent	SD	Pelt 44	Wood and Plastic	None	6	Yes	2020
	FI2	1.1–2.5	81–89	Normal	Detergent	SD	Pelt 44	Wood and Plastic	None	6	Yes	2020
	FI3	1.1–2.5	70–80	Normal	Bleach	GD	Pelt 44	Wood and Plastic	Bleach	6	Yes	2020
	S	0–1	90–95	Normal	Bleach	GD	Score	Wood and Plastic	Pelt44 + Vapor + Copper	10	Yes	2020
Zaida−Midelt	ASL	1.1–2.5	81–89	Normal	Detergent	GD	Pelt 44	Wood and Plastic	Detergent	8	No	2021
	Ml	1.1–2.5	81–89	Normal	Detergent	GD	None	Wood and Plastic	None	7	No	2021
	AH	1.1–2.5	81–89	Normal	Detergent	GD	None	Wood and Plastic	Detergent	7	No	2021
	DA	1.1–2.5	81–89	Normal	Pelt44	GD	None	Wood and Plastic	Detergent	6	No	2021
	Aby	0–1	90–95	Normal	Fumigation	GD	Pelt 44	Wood and Plastic	Fumigation	9	No	2021
	Tl	0–1	90–95	Normal	Bleach	GD	Score	Plastic	Bleach	10	No	2021
	BK	1.1–2.5	81–89	CA (O_2_: 2–3%, CO_2_: 2–3%)	Bleach	GD	Pelt 44	Plastic	Bleach	9	No	2021
	HM	1.1–2.5	81–89	Normal	Detergent	GD	None	Plastic	Detergent	6	No	2021
	TM	1.1–2.5	81–89	Normal	Detergent	GD	None	Plastic	None	8	Yes	2021
	FM1	1.1–2.5	90–95	Normal	Detergent	GD	Score	Plastic	Bleach	8	Yes	2021
	FM2	0–1	90–95	Normal	Bleach	GD/StD/Fuji	Pelt 44	Plastic	Bleach	9	Yes	2021
	FM3	0–1	81–89	Normal	Detergent	GD/StD/Fuji	Pelt 44	Plastic	Detergent	6	Yes	2021
	FM4	0–1	81–89	Normal	Detergent + Pelt44	GD	None	Plastic	Detergent	6	Yes	2021
	FM5	0–1	81–89	Normal	Bleach	GD	Pelt 44	Wood and Plastic	Bleach	8	Yes	2021
	FM6	1.1–2.5	90–95	Normal	Detergent + Pelt44	GD	Pelt 44	Wood and Plastic	Detergent + Pelt44	7	Yes	2021
	FM7	0–1	90–95	Normal	Detergent + Pelt44	GD	None	Plastic	None	8	No	2021
	FM8	0–1	90–95	Normal	Detergent	GD	None	Plastic	Detergent + Pelt44	8	Yes	2021
	FM9	0–1	81–89	Normal	Detergent	GD/StD/Fuji	Pelt 44	Plastic	Bleach	7	No	2021
	BD	0–1	90–95	Normal	Pelt44	StD	Score	Plastic	Soda	9	No	2021

CC: cold chamber; ST: storage temperature; RH: relative humidity; CCT: cold chamber type; CA: controlled atmosphere; CCD: cold chamber disinfection; AC: apple cultivar (GD: Golden Delicious, StD: Starking Delicious); PHT: postharvest treatment; BT: box type; BD: box disinfection; SD: storage duration; FSBS: fruit sorting before storage; RY: reference year.

## Data Availability

The data is contained within the manuscript and supplementary materials.

## Supplementary Materials

The following supporting information can be downloaded at https://www.mdpi.com/article/10.3390/plants13040553/s1. Table S1: Detailed information on fungal isolates that cause postharvest apple diseases identified by sequencing the partial β-tubulin gene; Table S2: Specific information on the β-tubulin and AMT4-EMR markers used in this investigation. Detailed PCR conditions can be found in the bibliographic references listed [79,80].
