# Supplementary material for: Unlocking Nature’s Secrets: Molecular Insights into Postharvest Pathogens Impacting Moroccan Apples and Innovations in the Assessment of Storage Conditions
Source: Plants (Basel). 2024 Feb 18;13(4):553. doi: 10.3390/plants13040553 (PMC10891559; doi:10.3390/plants13040553)
Supplement: Supplementary file 1 [file plants-13-00553-s001.zip › plants-2759362-supplementary.pdf]

Supplementary Materials

Table S1. Detailed information on fungal isolates that cause postharvest apple diseases identified by sequencing the partial β-tubulin gene.

Isolate code	Species	Sampling year	Origin	GPS coordinates	Accession number	Similarity percentage
**MY2**	*Neofabraea vagabunda*	2022	Meknes	N 33° 59' 36,8088'' W 5° 12' 2,4948''	OR753458	99,72% (OL740379)
**Aby4**	*Penicillium expansum*	2021	Midelt	N 32° 45' 2,2896'' W 5° 1' 45,1776''	OL802926	100% (MZ393463)

Table S2. Specific information on the β-tubulin and AMT4-EMR markers used in this investigation. Detailed PCR conditions can be found in the bibliographic references listed.

Partial gene name	Code of Primers	Sequence	Annealing T°	Reference
**β-tubulin**	Bt2a	GGTAACCAAATCGGTGCTGCTTTC	58	[79]
	Bt2b	ACCCTCAGTGTAGTGACCCTTGGC		
**AMT4-EMR**	AMT4-EMR-F	CTCGACGACGGTTTGGAGAA	66	[80]
	AMT4-EMR-R	TTCCTTCGCATCAATGCCCT		
